# The effects of solution-focused group therapy on peer friendship quality in adolescents with anxiety disorders

**DOI:** 10.3389/fpsyg.2026.1839700

**Published:** 2026-06-10

**Authors:** Qian Zhuang, Sinuo Zhang, Yanan Gu, Xueyun Wang, Cong Zhang, Bowen Zhang, Zhenghua Yang, Lu Li, Meng Li, Lixia Zhang

**Affiliations:** The Eighth People’s Hospital of Zhengzhou, Zhengzhou, China

**Keywords:** adolescents, anxiety disorders, peer friendship quality, peer relationships, solution-focused group therapy

## Abstract

**Objective:**

To explore the impact of Solution-Focused Group Therapy on peer friendship quality in adolescents with anxiety disorders.

**Methods:**

Eighty adolescent patients (aged 12–18) with anxiety disorders treated at the hospital outpatient department from September 2024 to October 2025 were selected. According to the principle of random allocation, they were divided into an experimental group and a control group. The control group received conventional medication and psychological interventions. The experimental group additionally received 8-week, short-term Solution-Focused Group Therapy (one 90-min session per week). Statistical analysis was performed using the Clinical Global Impression Scale (CGI), Hamilton Anxiety Scale (HAMA), 24-item Hamilton Depression Scale (HAMD-24), and Friendship Quality Questionnaire (FQQ) to assess patients’ mental health status and friendship quality before intervention (Week 0), after intervention (Week 8), and at an 8-week follow-up (Week 16).

**Results:**

Before the intervention, there were no statistically significant differences in scores between the groups (*p* > 0.05). After the intervention and at follow-up, HAMA, and HAMD-24 scores at each time point in the experimental group were significantly lower than those in the control group (*p*s < 0.05), the FQQ scores were significantly higher than those in the control group (*p*s < 0.05). CGI assessment confirmed that the overall efficacy in the experimental group was significantly higher than that in the control group (*p* < 0.001).

**Conclusion:**

Solution-Focused Group Therapy may effectively enhance peer friendship quality in adolescent patients with anxiety disorders and simultaneously alleviate their anxiety and depressive symptoms.

## Introduction

Adolescence is a critical stage for individual physical and mental development, accompanied by significant changes in physiology, psychology, and social relationships (Das et al., 2017; Powers et al., 1989). The developmental tasks of this period, such as establishing self-identity, developing independence, and building close peer relationships, present both opportunities for growth and substantial challenges (Paus et al., 2008). Data from around the world indicate that adolescents are facing an escalating mental health crisis (Roach, 2018), with anxiety disorders having become one of the most prevalent and debilitating issues (Ko et al., 2024; Resch and Parzer, 2024).

Anxiety disorders are typically characterized by excessive, uncontrollable worry and fear, often accompanied by physical symptoms such as palpitations, sweating, and muscle tension (Szuhany and Simon, 2022). In adolescents, anxiety may manifest in various forms, including generalized anxiety, social anxiety, separation anxiety, or panic attacks, significantly disrupting their daily academic and social functioning (Tng et al., 2025).

Epidemiological surveys indicate a high prevalence of anxiety among adolescents (Kramer and Francis, 2025). Data from the World Health Organization show that a significant proportion of adolescents aged 10–19 worldwide are affected by anxiety disorders, with the incidence showing an increasing trend year by year. This poses profound negative impacts on adolescents’ cognitive functioning, academic achievement, and everyday life (Seidl et al., 2021).

Within the framework of the social ecological systems theory of adolescence, peer relationships—particularly the quality of friendships—play a critical role (Roach, 2018). High-quality peer friendships provide adolescents with emotional support, social companionship, and a sense of identity, serving as important protective factors in coping with stress and developing social competencies (Sharpe et al., 2014; Telzer et al., 2015). However, anxiety symptoms often severely undermine this protective buffer. A growing body of research has demonstrated that anxious adolescents, particularly those presenting with social anxiety, exhibit marked impairments in friendship quality, manifesting as diminished intimacy, reduced companionship, compromised conflict resolution capacities, and elevated interpersonal distress (Rodebaugh et al., 2015; Scharfstein et al., 2011). Propelled by a pervasive fear of negative social evaluation, these adolescents are predisposed to avoid social contexts, withdraw from peer engagement, and demonstrate deficiencies in the social-cognitive and behavioral competencies requisite for the initiation and maintenance of meaningful friendships (Mehtalia and Vankar, 2004). The ensuing poor-quality peer relationships—characterized by social isolation, interpersonal conflict, and, in some cases, peer victimization—operate as a chronic interpersonal stressor that further exacerbates anxiety symptomatology, thereby establishing a self-perpetuating cycle between anxiety and peer relational dysfunction (Liu et al., 2022). Disrupting this reciprocal cycle, accordingly, necessitates intervention approaches that extend beyond symptom alleviation alone and directly target the social-relational domain.

At present, interventions for adolescent anxiety disorders primarily focus on individual-level approaches such as cognitive-behavioral therapy or medication, aiming to directly alleviate core symptoms (Liu et al., 2025; Zelviene et al., 2023). Existing research has demonstrated that CBT can reliably reduce anxiety symptoms in adolescents and produce moderate improvements in their social functioning and peer relationships (Etkin et al., 2023). However, owing to substantial heterogeneity across intervention modalities and study samples, it remains difficult to draw definitive conclusions regarding which specific treatments improve which specific outcomes. Nevertheless, merely concentrating on symptom reduction at the individual level may be insufficient to break the aforementioned vicious cycle (Tang et al., 2021). Expanding the scope of intervention to the social relational level, particularly by targeting the enhancement of peer friendship quality, holds significant clinical relevance and practical necessity.

Group therapy, by contrast, may help to address this gap. Unlike individual treatment, group-based interventions afford an *in vivo* social milieu in which interpersonal processes can be directly engaged (Arias-Pujol and Anguera, 2017). Such a setting provides adolescents with a developmentally salient context for consolidating their personal, social, and sexual identities. Concurrently, the group facilitates the integration of past, present, and future experiences, thereby fostering an awareness of novel behavioral possibilities within peer relationships (Leader, 1991). For adolescents whose anxiety is sustained by avoidance of socially evaluative contexts, the group itself functions simultaneously as a therapeutic medium and as a graduated, controlled exposure environment, enabling skill acquisition and the cultivation of supportive peer bonds to proceed in tandem. Solution-Focused Brief Therapy (SFBT), as a postmodern psychotherapeutic paradigm, centers on identifying individuals’ strengths, resources, and future possibilities for resolving issues, rather than focusing on the causes of problems (Franklin et al., 2017). Applying this approach in a group setting provides a unique intervention platform for adolescents with anxiety disorders. Within the group, members can not only learn solution-focused techniques to set specific, positive social goals but also directly practice social skills, receive feedback and encouragement from peers, and restructure their cognitions about themselves and interpersonal relationships in a safe and supportive environment (Nickerson, 1995). Currently, Solution-Focused Group Therapy (SFGT) has been widely used in interventions for patients with various conditions such as depression, anxiety, insomnia, substance use and cancer (Carr et al., 2014; Liu et al., 2015; Vermeulen-Oskam et al., 2024; Walker et al., 2022; Żak and Pêkala, 2025; Zhang et al., 2018).

In parallel, a growing body of research has demonstrated that SFBT yields significant effects in alleviating anxiety and depressive symptoms among adolescents (Chen et al., 2023). With respect to relational outcomes, a recent meta-analysis by Franklin et al. further reported that SFBT confers moderate improvements in psychosocial adjustment and family functioning (Franklin et al., 2023), suggesting that this therapeutic orientation holds preliminary potential for enhancing interpersonal functioning. Given that improvements in psychosocial adjustment and family functioning often co-occur with changes in peer relationships, these findings hint at a possible link between SFBT and friendship quality, yet direct evidence remains absent. Nevertheless, controlled trial evidence examining the effects of SFGT on peer friendship quality among adolescents with anxiety disorders remains markedly scarce.

The present study seeks to address this gap by examining the effects of SFGT, delivered as an adjunct to treatment as usual (TAU), on both anxiety symptoms and peer friendship quality among Chinese adolescents diagnosed with anxiety disorders. Drawing on a social-ecological framework, we hypothesized that, relative to the symptom-level effects of TAU alone, the addition of SFGT would (a) yield further reductions in anxiety symptoms, and (b) produce greater improvements in peer friendship quality. We further hypothesized that gains in peer friendship quality would be sustained at the 16-week follow-up assessment, thereby supporting the proposed mechanism of action: that intervening at the social-relational levelhereby supporting the proposed mechanism of action: that intervening a self-efficacyal levelhereby supporting the proposed mechanism of action: thtom-focused TAU and helps interrupt the reciprocal cycle between anxiety symptoms and peer-relational difficulties.

## Materials and methods

### Study participants

All participants were recruited from the outpatient department of the Eighth People’s Hospital of Zhengzhou (Zhengzhou Mental Health Center). This hospital is a municipal-level specialized psychiatric institution that undertakes the diagnosis and treatment of mental and psychological disorders, as well as research and teaching functions within the region. All participants sought treatment on their own initiative for anxiety-related concerns and received standard outpatient care following assessment by a psychiatrist. A total of 112 adolescents with mild to moderate anxiety disorders who sought treatment at the Eighth People’s Hospital of Zhengzhou from November 2024 to October 2025 were initially screened for eligibility. After applying the inclusion and exclusion criteria, 32 patients were excluded, including 11 who did not meet the diagnostic criteria, 21 who declined to participate, and none for other reasons. The remaining 80 eligible participants were enrolled and randomly allocated into an experimental group (*n* = 40) and a control group (*n* = 40) using a random number table method. Due to the extended duration of the study and the inherent characteristics of patients with psychological disorders, some participants dropped out during the trial. Ultimately, valid data were collected from 31 cases in the experimental group and 33 cases in the control group, with discontinued participation rates of 22.5 and 17.5%, respectively. The participant flow diagram following the CONSORT 2010 statement is presented in Supplementary Appendix.

Inclusion criteria: (1) Age 12 cr years. (2) Meeting ICD-10 diagnostic criteria of anxiety disorder, confirmed by two attending psychiatrists through independent assessment, with uncertain cases resolved through research team discussion and consensus. (3) Score on the Hamilton Anxiety Scale (HAMA) ≥ 14. (4) Education level of primary school or above. (5) Informed consent obtained from both the patient and their legal guardian.

Exclusion criteria: (1) Current or previous comorbid major psychiatric disorders. (2) Presence of severe physical illnesses. (3) Current or past history of substance dependence or abuse, including tobacco or alcohol use. (4) History of suicide attempts (self-injurious behavior with intent to end life). (5) Concurrent participation in other studies or refusal to participate in this study.

This study was reviewed and approved by the hospital’s ethics committee, and informed consent was obtained from all patients or their legal guardians.

This study complies with the Declaration of Helsinki and has been approved by the Institutional Ethics Committee of The Eighth People’s Hospital of Zhengzhou (Approval No.: 2024-KY-005). Written informed consent was obtained from all participants.

### Methods

Treatment for the experimental group included conventional treatment—comprising medication guidance by physicians, physical therapy, health education on disease knowledge, emotional nurse-patient interactions, and family emotional supportrapy, heal regular weekly sessions of Solution-Focused Group Therapy (SFGT). A professional intervention team was established, consisting of doctors and psychotherapists. The team included 4 chief psychiatrists and 7 psychotherapists, responsible for designing, implementing, and evaluating the SFGT intervention protocol. Each group comprised 6. Each group comprts. The team included 4 -level psychotherapists (certified in SFGT with over 5 years of group facilitation experience) and supervised throughout by one attending psychiatrist. The control group also received pharmacotherapy and physical therapy under medical guidance and participated in a weekly 90-minute non-interactive session, which involved no group-based intervention. These gatherings were facilitated by the same intervention team mentioned above. Throughout the 90-min gathering, participants were free to engage in self-directed activities within the group activity room, such as completing homework, chatting with other members, reading, or similar individual or informal social activities. This design was intended to control for non-specific therapeutic factors, such as regular visits to the hospital, contact with staff, and the socialization and placebo effects arising from being in the same room with other patients. By providing structured time while removing the core group therapeutic techniques, we were able to more clearly attribute the effects observed in the experimental group to Solution-Focused Group Therapy itself, rather than to general group interaction or attention.

The experimental group formed a total of 6 SFGT groups (comprising 3 groups of 6 members, 2 groups of 7 members, and 1 group of 8 members; the 40 participants were assigned to each group according to their time of enrollment. The control group concurrently formed 6 corresponding non-interactive gathering groups with the same group sizes). Thus, a total of 12 groups were run in this study.

To ensure intervention fidelity, the following measures were implemented: (1) All group therapists held intermediate-level or higher national certifications in psychotherapy, had completed systematic training in Solution-Focused Brief Therapy and group facilitation, and possessed at least 5 years of group therapy experience. (2) The intervention strictly adhered to the Standardized Manual for Solution-Focused Group Therapy for Adolescent Anxiety Disorders, developed by our research team. This manual was refined through two rounds of expert consultation and protocol revision involving 20 experts from the fields of psychiatry, clinical psychology, and education, employing a Delphi method approach and tailored to the high-pressure educational context of central China. Each session was structured with specific themes, objectives, step-by-step procedures, and core question examples. (3) Throughout the study period, after the completion of each group session, group members were asked to complete a group satisfaction questionnaire. Two senior therapists who were not involved in leading that particular group then evaluated the feedback questionnaires, together with debriefing interviews conducted with the group facilitators. The frequency and quality with which core SFBT techniques (such as exception questions, miracle questions, scaling questions, and compliments) were applied were rated and used to provide feedback, thereby ensuring intervention adherence and quality.

The Solution-Focused Brief Group Therapy (SFGT) intervention protocol designed for this study was structured to be conducted once per week over a total of 8 sessions, with each session lasting 90 min. All groups were closed-ended; that is, once a group commenced, no new members were admitted, thereby ensuring a sense of safety and group cohesion and facilitating an effective intervention process. The entire therapeutic process was organized into three core phases according to the logical progression of group intervention: The first phase was the Establishment Phase, corresponding to sessions 1ase, corresponding oup interventionan effectivto foster the development of trust among group members, enhance overall group cohesion, and assist members in clearly defining specific intervention goals—such as “improving active expression skills during interpersonal interactions”—thereby laying a solid foundation for subsequent intervention. The second phase was the Working Phase, encompassing sessions 3–7. This phase focused on applying core techniques of Solution-Focused Brief Therapy. Through guided questioning, members were supported in exploring their latent resources. For example, exception questions were used to prompt reflection: “Can you recall past experiences where you successfully communicated effectively with classmates?” Miracle questions were employed to encourage envisioning future possibilities: “If your interpersonal relationships improved significantly, what changes would you notice in your daily life?” Building on these reflections, members were further assisted in formulating actionable and concrete plans for change, promoting the gradual implementation of these changes in real-life contexts. The third phase was the Termination Phase, corresponding to session 8. The main tasks of this phase were to systematically summarize the progress achieved by members throughout the intervention, reinforce their internal motivation to sustain changes, and, through targeted guidance and support, help alleviate potential feelings of guilt or anxiety related to the conclusion of the group. This ensured the continuity of intervention effects and achieved a coherent closure of the therapeutic process.

A detailed overview of the eight-session SFGT protocol, including session themes, main activities, and core solution-focused techniques, is provided in Supplementary Appendix.

### Outcome measures and evaluation criteria

Before patients participated in the group therapy (pre-treatment, Week 0), at the completion of the 8th session (post-treatment, Week 8), and during follow-up (follow-up, Week 16), psychiatric medical staff with intermediate or higher professional titles, who had received uniform training, conducted assessments using standardized instructions. The evaluators will remained blinded to the group assignments. After each assessment, the evaluator was asked to guess the participant’s group assignment. A chi-square test revealed no significant association between the evaluators’ guesses and the actual group assignments (χ^2^ = 0.05, *p* = 0.823), suggesting that blinding was maintained to a reasonable degree.

### Clinical efficacy evaluation

The Hamilton Anxiety Scale (HAMA) (Maier et al., 1988), the 24-item Hamilton Depression Scale (HAMD-24) (Fenton and McLoughlin, 2021), and the Clinical Global Impression (CGI) (Busner and Targum, 2007) questionnaire were used to assess patients’ psychological status and clinical outcomes.

The HAMA consists of 14 items, with a total score ranging from 0 to 56, which effectively reflects the severity of anxiety symptoms. A score above 7 may indicate possible anxiety. The scale demonstrates good reliability and validity, with a Cronbach’s α coefficient of 0.922.

The HAMD-24 includes 24 items, and its total score reliably reflects the severity of depression. A score above 8 may suggest possible depression. The scale also shows good reliability and validity, with a Cronbach’s α coefficient of 0.902.

The Clinical Global Impression (CGI) questionnaire was employed to evaluate clinical efficacy. It comprises three components: Severity of Illness (SI), Global Improvement (GI), and Efficacy Index (EI). Both SI and GI are scored on a scale of 0 to 7, with higher SI scores indicating greater illness severity and lower GI scores reflecting more significant and favorable changes. The EI is calculated as the therapeutic effect score divided by the side effect score, with higher values indicating better overall efficacy.

### Peer friendship quality

In this study, the Friendship Quality Questionnaire (FQQ) revised by Zongkui et al. (2006) was adopted. This instrument was selected for three main reasons. First, it is one of the few friendship quality measures specifically validated for Chinese child and adolescent populations. Second, its abbreviated 18-item version reduces respondent burden while retaining good psychometric properties. Third, its three-factor structure captures both positive and negative dimensions of friendship, which is particularly relevant for understanding the social difficulties associated with adolescent anxiety. The scale was originally developed by Parker and Asher (1993) to assess the quality of friendship with one’s best friend. In 1998, Zou et al. (1998) adapted the questionnaire to suit the Chinese context. This abbreviated version was revised by Zongkui et al. (2006) based on the original 40-item questionnaire, retaining the three dimensions with the highest factor loadings (Companionship and Recreation, Conflict and Betrayal, and Help and Guidance), comprising a total of 18 items, serving as a short form of the 40-item Friendship Quality Questionnaire. After reverse-scoring the items related to conflict and betrayal, the scores of all 18 items are summed to obtain a total friendship quality score. Higher total scores indicate better friendship quality. This short version has been widely used among Chinese adolescent populations, in both clinical and non-clinical samples. Hu et al. reported a Cronbach’s α coefficient of 0.83 in a large-sample study of Chinese adolescents (Hu et al., 2025), and Yang et al. further verified its good psychometric properties in a sample of Chinese children (Yang et al., 2025). In the present study, the scale’s Cronbach’s α coefficient was 0.827, consistent with previous reports, supporting its applicability in a clinical population.

### Statistical methods

Statistical analysis was performed using SPSS 22.0 software. Continuous data were expressed as mean ± standard deviation (M ± SD). Within-group comparisons before and after the intervention were analyzed using paired *t*-tests, while between-group comparisons were conducted using independent samples *t*-tests. Comparisons across multiple time points were performed using repeated measures analysis of variance (with analysis of main effects and interaction effects). Categorical data were presented as rates, and between-group comparisons were conducted using the chi-square test. A *p*-value of < 0.05 was considered statistically significant.

## Results

### Baseline data of the two groups

A total of 80 adolescent patients with anxiety were recruited for this study, with complete data collected from 64 participants included in the final analysis. Among them, the experimental group comprised 31 patients (12 males, 19 females) with a mean age of 15.29 ± 1.75 years, while the control group consisted of 33 patients (12 males, 21 females) with a mean age of 15.36 ± 1.61 years. Among the experimental group, 24 participants (77.4%) were from urban areas and 7 (22.6%) from rural areas, while in the control group, 22 (66.7%) were from urban areas and 11 (33.3%) from rural areas. Regarding only-child status, 12 participants (38.7%) in the experimental group were only children and 19 (61.3%) were not, whereas in the control group, 9 (27.3%) were only children and 24 (72.7%) were not. No statistically significant differences were observed in baseline characteristics between the two groups (*p*s > 0.05) (see Table 1). A comparison of baseline data between treatment completers (*n* = 64) and dropouts (9 from the experimental group, 7 from the control group) revealed no statistically significant differences in age, gender composition, or baseline HAMA, HAMD-24, and FQQ scores (ps > 0.05), suggesting that the attrition may have been random in nature (see Table 2).

**TABLE 1:** Comparison of baseline data between the two groups of adolescents with anxiety disorder [M ± SD, n (%)].

Group	Gender [*n* (%)]		Household registration [*n* (%)]		Only child [*n* (%)]		Age (years, M ± SD)
	Male	Female	Urban	Rural	Yes	No	
Experimental group	12 (38.7)	19 (61.3)	24 (77.4)	7 (22.6)	12 (38.7)	19 (61.3)	15.29 ± 1.75
Control group	12 (36.4)	21 (63.6)	22 (66.7)	11 (33.3)	9 (27.3)	24 (72.7)	15.36 ± 1.61
*t*/χ^2^	0.038		0.914		0.948		0.174
*p*	0.846		0.339		0.330		0.689

**TABLE 2:** Comparison of baseline data between the completers and dropouts [M ± SD, *n* (%)].

Group	Gender [*n* (%)]		Age (years, M ± SD)	HAMA (week 0)	HAMD-24 (week 0)	FQQ (week 0)
	Male	Female				
Completers	24 (37.5)	40 (62.5)	15.33 ± 1.67	49.02 ± 10.38	34.97 ± 4.58	51.14 ± 11.03
Dropouts	6 (37.5)	10 (62.5)	15.50 ± 1.83	48.56 ± 10.51	35.94 ± 4.02	51.13 ± 9.95
*t*/χ^2^	0.000		-0.361	0.156	-0.774	0.005
*p*	1.000		0.719	0.877	0.441	0.996

### Analysis of the clinical global impression (CGI) results

Repeated measures ANOVA was applied for SI. Since the assumption of sphericity was violated across time points (*p* = 0.023), the Greenhouse–Geisser correction was used. The results showed a significant main effect of time, *F*(1.791, 111.028) = 102.555, *p* < 0.001, *η^2^* = 0.623; a significant main effect of group, *F*(1, 62) = 16.450, *p* < 0.001, *η^2^* = 0.210; and a significant time × group interaction, *F*(1.791, 111.028) = 33.311, *p* < 0.001, *η^2^* = 0.349. Further simple effects analysis revealed that at the pre-treatment, the group effect was not significant (*p* = 0.822). At both the post-treatment and follow-up, SI scores decreased significantly (*p* < 0.001). Within the experimental group, SI scores differed significantly between pre-treatment and post-treatment (*p* < 0.001), pre-treatment and follow-up (*p* < 0.001), and post-treatment and follow-up (*p* = 0.007). In the control group, SI scores at post-treatment and follow-up were significantly lower than at pre-treatment (*p* = 0.001), but no significant difference was observed between post-treatment and follow-up (*p* = 0.729).

Independent samples *t*-tests were conducted for Global Improvement (GI) and Efficacy Index (EI), with the results presented in Table 3. The GI scores demonstrated better therapeutic effects in both the post-treatment and follow-up stages. For EI, there was no significant difference between the experimental and control groups at the post-treatment stage, but by the follow-up stage, the scores had increased significantly, indicating an enhanced level of therapeutic efficacy.

**TABLE 3:** Comparisons of CGI scores between the two groups of adolescents with anxiety disorder at two/three time points (M ± SD).

Group	SI			GI		El	
	Week 0	Week 8	Week 16	Week 8	Week 16	Week 8	Week 16
Experimental group	3.87 ± 0.81	2.45 ± 0.72^a^	2.03 ± 0.71^a,b^	2.35 ± 0.71	1.94 ± 0.77 ^b^	1.94 ± 0.60	2.5 ± 0.77 ^b^
Control group	3.82 ± 1.04	3.45 ± 0.83^a^	3.33 ± 0.81^a,b^	3.27 ± 0.76	2.94 ± 0.70 ^b^	1.95 ± 0.80	1.86 ± 0.88 ^b^
*t*	-	-	-	-4.98	-5.44	-0.135	3.068
*p*	0.822	<0.001	<0.001	<0.001	<0.001	0.892	0.003

^a^*p* < 0.05 compared with scores at week 0 pre-treatment.

^b^*p* < 0.05 compared with scores at week 8 post-treatment within the same group.

### Comparisons of HAMA, HAMD-24, and FQQ scores between the two groups at three time points

Repeated measures ANOVA was employed for the HAMA scores (see Table 4). As the assumption of sphericity was violated across time points (*p* < 0.05), the GreenhousetGeisser corrected values were used. The results showed a significant main effect of time, *F*(1.329, 82.372) = 246.321, *p* < 0.001, *η^2^* = 0.799; a significant main effect of group, *F*(1, 62) = 19.820, *p* < 0.001, *η^2^* = 0.242; and a significant time × group interaction, *F*(1.329, 82.372) = 47.695, *p* < 0.001, *η^2^* = 0.435. Further simple effects analysis revealed that within both the experimental group and the control group, the differences across the three time points (pre-treatment, post-treatment, and follow-up) were statistically significant (*p*s < 0.001). At pre-treatment, there was no significant difference between the experimental and control groups (*p* = 0.496). However, at post-treatment, a statistically significant difference emerged between the two groups (*p* < 0.001), with the experimental group showing a significantly greater reduction in HAMA scores. This trend persisted at the 16-week follow-up (*p* < 0.001).

**TABLE 4:** Comparisons of HAMA, HAMD-24, and FQQ scores between the two groups of adolescents with anxiety disorder at two/three time points (M ± SD).

Group	Time	HAMA	HAMD-24	FQQ
Experimental group (*n* = 31)	Week 0	49.94 ± 9.34	38.26 ± 4.27	51.77 ± 10.65
	Week 8	24.68 ± 7.98^a,c^	25.42 ± 3.47^a,c^	61.58 ± 9.88^a,c^
	Week 16	20.45 ± 5.18 ^a,b,c^	22.65 ± 3.32 ^a,b,c^	67.61 ± 9.21^a,b,c^
Control group (*n* = 33)	Week 0	48.15 ± 11.34	31.88 ± 1.98	50.55 ± 11.51
	Week 8	39.21 ± 11.92^a^	26.85 ± 2.09^a^	52.67 ± 11.94^a^
	Week 16	36.09 ± 10.29 ^a,b^	24.45 ± 2.90^a,b^	53.30 ± 12.20^a^

^a^*p* < 0.05 compared with scores at week 0 pre-treatment within the same group.

^b^*p* < 0.05 compared with scores at week 8 post-treatment within the same group.

^c^*p* < 0.05 compared with the control group at the same time point.

Repeated measures ANOVA was conducted for the HAMD-24 scores. Since the assumption of sphericity was violated across time points (*p* = 0.041), the Greenhouse–Geisser correction was applied. The results indicated a significant main effect of time, *F*(1.819, 112.791) = 499.379, *p* < 0.001, *η^2^* = 0.890; no significant main effect of group, *F*(1, 62) = 2.732, *p* = 0.103, *η^2^* = 0.042; and a significant time × group interaction, *F*(1.819, 112.791) = 73.005, *p* < 0.001, *η^2^* = 0.541. Further simple effects analysis revealed that at pre-treatment, there was a statistically significant difference in HAMD-24 scores between the experimental group and the control group (*p* < 0.001), with the experimental group scoring higher (38.26) than the control group (31.879). At post-treatment, the experimental group’s score (25.42) had become significantly lower than that of the control group (26.85) (*p* = 0.049), and this trend remained at the 16-week follow-up (*p* = 0.023).

Given the observed baseline imbalance in HAMD-24 scores between the two groups, an analysis of covariance (ANCOVA) was further conducted with baseline HAMD-24 scores as the covariate to verify the robustness of the treatment effect. The assumption of homogeneity of regression slopes was met at both post-treatment [Group × HAMDpre: *F*(1, 60) = 0.645, *p* = 0.425] and follow-up (*p* = 0.079). After adjusting for baseline scores, the experimental group demonstrated significantly lower depressive symptoms than the control group at Week 8 [*F*(1, 61) = 25.010, *p* < 0.001, η^2^ = 0.291], with adjusted means of 23.94 (SE = 0.54) versus 28.24 (SE = 0.52). This significant difference was maintained at the 16-week follow-up [*F*(1, 61) = 18.595, *p* < 0.001, η^2^ = 0.234], where the adjusted means were 21.35 (SE = 0.63) for the experimental group and 25.67 (SE = 0.60) for the control group. These results confirm that the improvement in depressive symptoms in the experimental group remained robust even after controlling for the initial between-group disparity. Therefore, it is important to note that the difference observed between the two groups at pre-treatment was not due to selection bias.

Repeated measures ANOVA was conducted for the FQQ scores. The results showed a significant main effect of time, *F*(2, 124) = 51.829, *p* < 0.001; a significant main effect of group, *F*(1, 62) = 10.376, *p* < 0.001; and a significant time × group interaction, *F*(2, 124) = 25.235, *p* < 0.001. Further simple effects analysis revealed that at pre-treatment, there was no significant difference between the experimental group and the control group (*p* = 0.660). At post-treatment, a significant difference emerged (*p* = 0.002), and this difference remained significant at follow-up (*p* < 0.001). Specifically, the FQQ scores of the experimental group gradually increased across pre-treatment, post-treatment, and follow-up assessments, indicating a progressive improvement in friendship quality among participants in this group. In contrast, no significant differences were observed across the three time points in the control group, suggesting no notable enhancement in their friendship quality.

## Discussion

This study aimed to explore the intervention effect of Solution-Focused Group Therapy on peer friendship quality in adolescents with anxiety disorders. The results indicated that adding an 8-week Solution-Focused Group Therapy to conventional treatment not only significantly alleviated anxiety and depressive symptoms in adolescents but also effectively enhanced their perceived friendship quality. Furthermore, the therapeutic effects were maintained 8 weeks after the intervention ended. These findings support our research hypothesis and provide a feasible intervention pathway for breaking the “symptoms-relationships” vicious cycle in adolescents with anxiety disorders.

**FIGURE 1:**
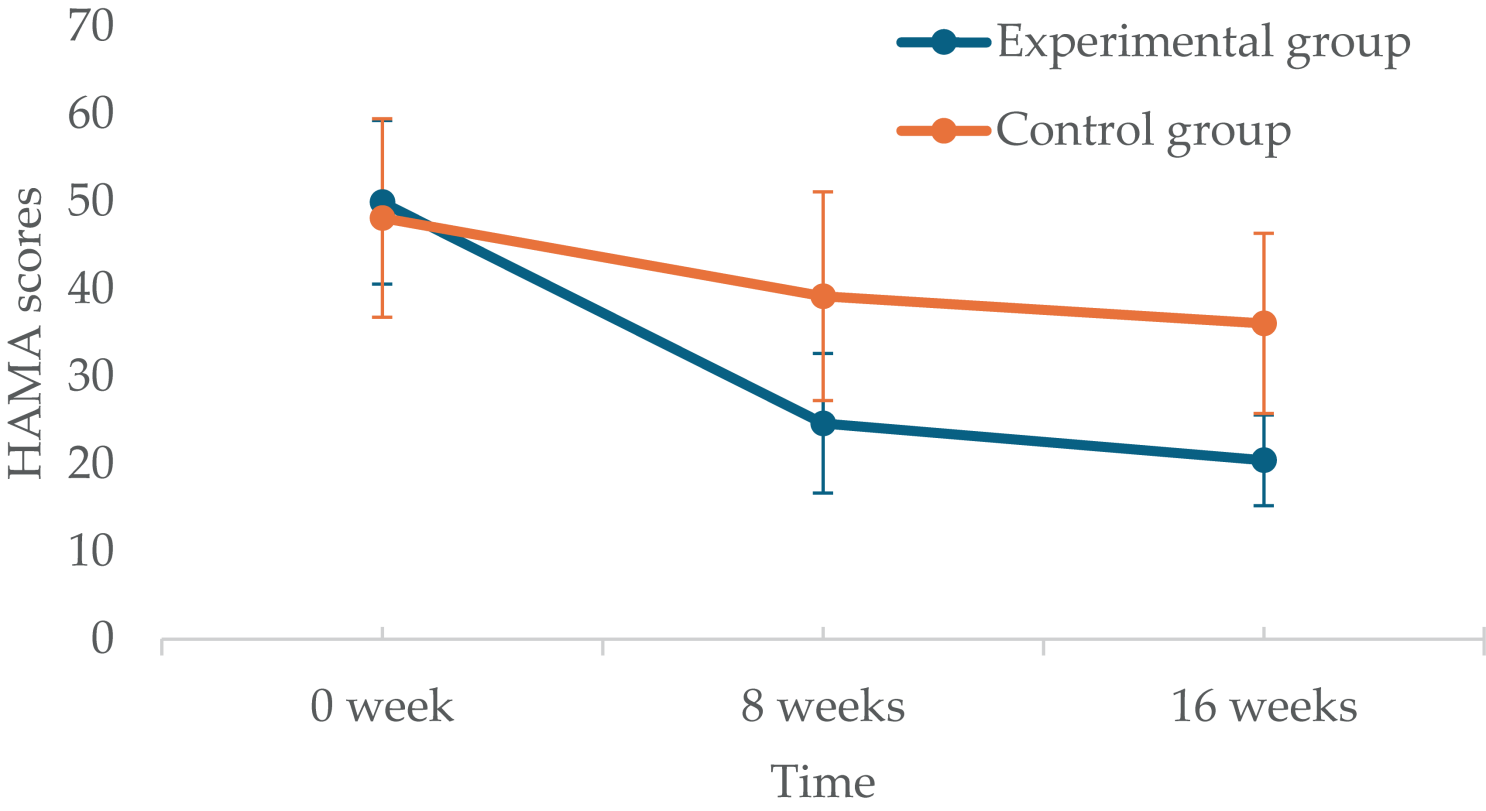
Trends in mean HAMA scores between the experimental and control groups across three time points: pre-treatment (Week 0), post-treatment (Week 8), and follow-up (Week 16).

**FIGURE 2:**
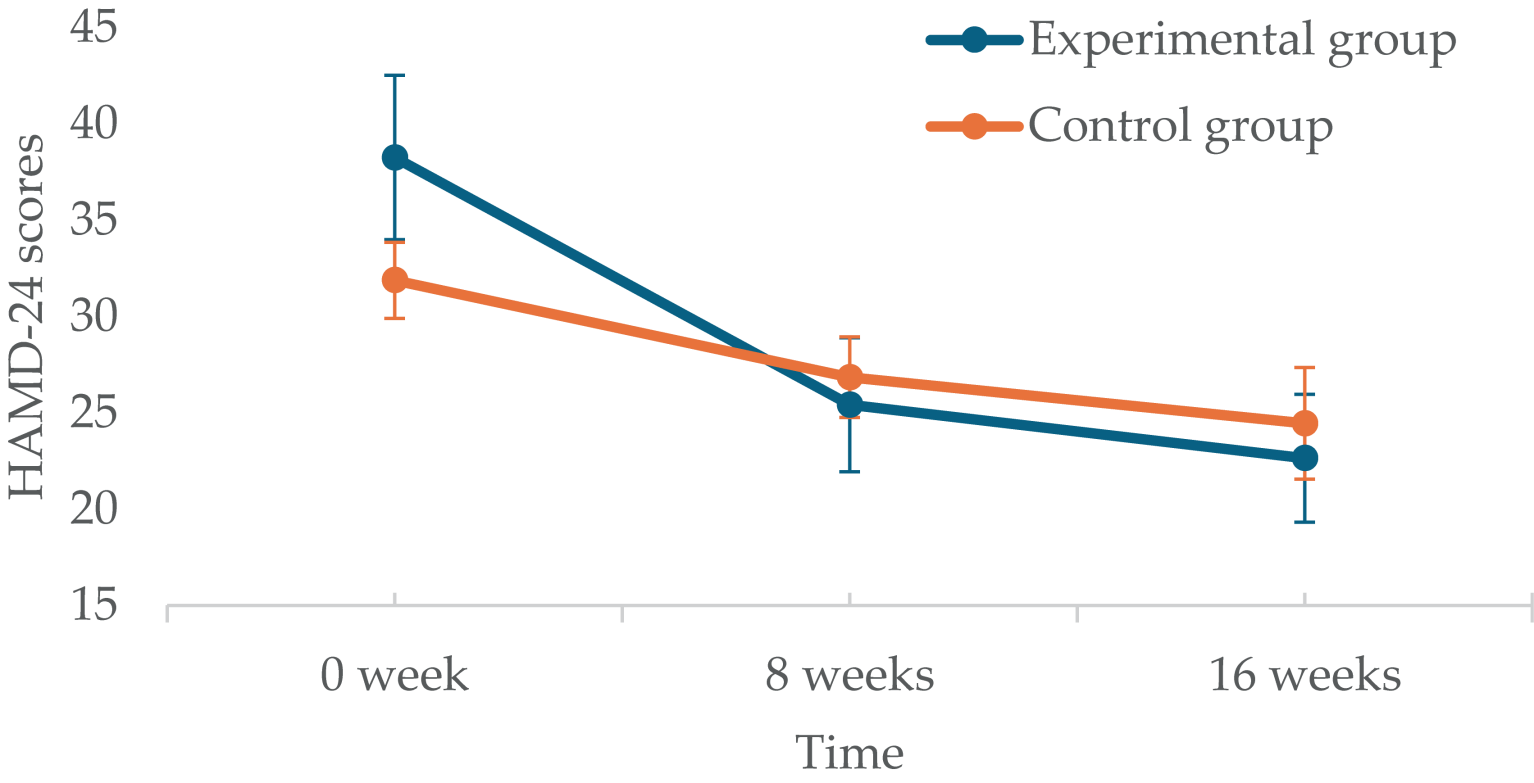
Trends in mean HAMD-24 scores between the experimental and control groups across three time points: pre-treatment (Week 0), post-treatment (Week 8), and follow-up (Week 16).

Adolescent patients in both groups showed significantly lower scores on the Hamilton Anxiety Scale (HAMA) and the Hamilton Depression Scale (HAMD-24) after 8 weeks of SFGT treatment and at the 16-week follow-up compared to their pre-treatment scores (see Figures 1, 2). These results indicate that both the conventional treatment alone and the combined intervention of SFGT with conventional treatment were effective in alleviating depressive and anxiety symptoms in adolescents that both the conventional treatment alone and the (Chen et al., 2024; Duan et al., 2023; Foo et al., 2024; Gingerich and Eisengart, 2000).

**FIGURE 3:**
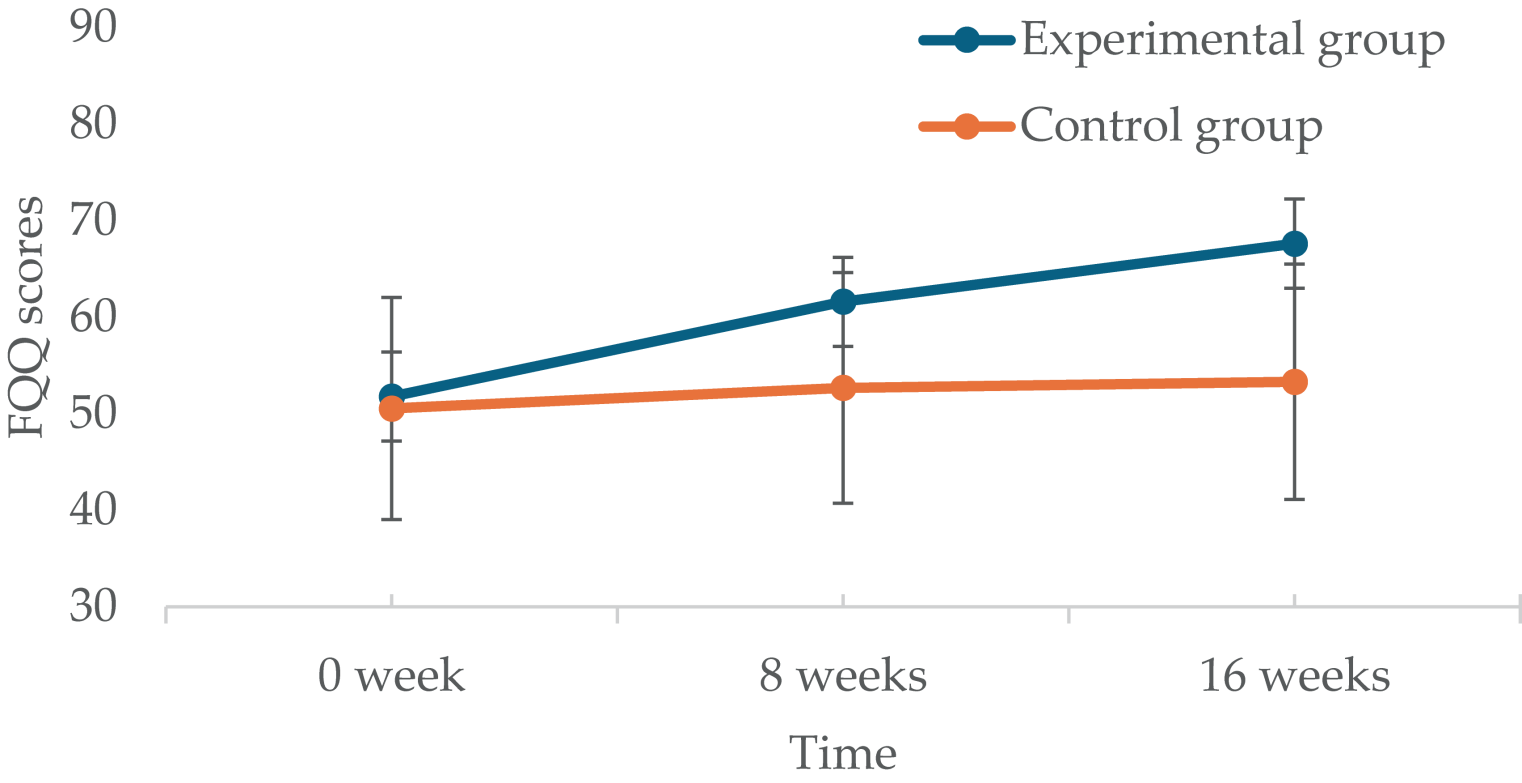
Trends in mean FQQ scores between the experimental and control groups across three time points: pre-treatment (Week 0), post-treatment (Week 8), and follow-up (Week 16).

The most important finding of this study lies in the fact that SFGT not only alleviates emotional symptoms but also directly promotes the development of positive peer relationships (see Figure 3). Compared with the control group, the continuous improvement in FQQ scores in the experimental group indicates that SFGT, through its structured group process, provides adolescents with real-life social practice scenarios and a positive feedback mechanism, thereby enhancing their real-world social competence and confidence (Chen and Lin, 2024). This result aligns with the perspective of “relationships as a vehicle for psychological recovery” in social ecological systems theory and highlights the clinical necessity of shifting from symptom-focused interventions alone to a “symptom-relationship” dual-focus intervention approach.

Furthermore, through a multi-time-point tracking design, this study reconfirmed the stable short- and medium-term effects of SFGT (Gong and Hsu, 2017; Pu et al., 2023). Compared with traditional problem-centered therapeutic approaches, Solution-Focused Brief Therapy places greater emphasis on identifying individual resources and constructing future visions, and can avoid the psychological burden that may arise from repeatedly dissecting problems. In theory, this aligns well with the developmental needs of adolescents who are in the process of establishing self-identity and building peer relationships (Carrera et al., 2016). Additionally, this study conducted group comparisons while controlling for conventional treatment, which enhances the comparability and persuasiveness of the results.

It is worth noting that the significant baseline difference in HAMD-24 scores between the two groups, with the experimental group presenting a higher initial depressive symptom burden, likely reflects the natural clinical heterogeneity in the severity of comorbid depression among adolescents with anxiety disorders, rather than a failure of randomization or systematic selection bias. Importantly, the ANCOVA results demonstrated that SFGT yielded robust and sustained improvements in depressive symptoms even after controlling for this initial disparity, indicating a genuine treatment effect rather than an artifact of baseline differences. A plausible mechanism is that SFGT may indirectly enhance emotional regulation by boosting individuals’ self-efficacy and social competence through structured group processes, thereby generating therapeutic momentum that transcends initial symptom severity.

Regarding the Clinical Global Impression Efficacy Index (CGI-EI), a noteworthy pattern emerged: no significant between-group difference was observed at Week 8, whereas a marked advantage in the experimental group became apparent at the 16-week follow-up. This delayed emergence of improvement may reflect a core characteristic of psychosocial interventions—the acquisition, internalization, and real-life transfer of interpersonal and cognitive skills require a gradual developmental process that extends beyond the period of active intervention. Conventional treatment primarily targets rapid symptomatic relief, with relatively limited sustained impact on broader psychosocial functioning. In contrast, the resource-oriented thinking and proactive social competencies cultivated through structured SFGT sessions become consolidated as internalized personal assets. When routine medical and supportive interventions are gradually tapered off, these ingrained abilities continue to exert their empowering effects, eventually driving a significant divergence in overall functional recovery at follow-up. This finding highlights the importance of incorporating longer-term follow-up assessments when evaluating the efficacy of psychosocial interventions such as SFGT, as their full benefits in terms of functional recovery may take time to manifest fully.

The findings of this study offer relatively clear practical implications for adolescent mental health services. First, a structured 8-week Solution-Focused Group Therapy protocol can serve as an effective adjunct to existing routine outpatient treatment, particularly when addressing social isolation and interpersonal difficulties that are closely intertwined with anxiety. Second, because this approach focuses on resources rather than pathology, it can foster a non-judgmental, de-stigmatizing peer environment, which is especially appealing to adolescents with anxiety who tend to be highly sensitive to evaluation. These insights encourage clinicians to set dual goals—“symptom relief” and “social functioning enhancement”—when developing intervention plans, and to utilize group-based psychological interventions as a bridge connecting individual internal change with external social adaptation.

The present findings also raise the question of through what mechanisms SFGT may improve peer friendship quality. Although the current study was not designed to formally test mediation, several plausible pathways merit discussion. First, SFGT may enhance self-efficacy in social situations. Core SFBT techniques—such as exception questions and scaling questions—guide adolescents to recognize their often overlooked interpersonal strengths and past successes. This repeated reinforcement may gradually rebuild social self-efficacy, which is frequently diminished by chronic anxiety, thereby promoting approach behaviors and creating more opportunities for positive peer interactions (Bandura, 1997). Second, the group setting itself serves as a “social microcosm,” offering a safe environment for *in vivo* practice of social skills. Members receive real-time peer feedback and therapist reinforcement through techniques such as complimenting, which may facilitate the generalization of these skills to real-world relationships. Third, the alleviation of anxiety and depressive symptoms may act as a facilitative condition: as emotional distress subsides, adolescents become more willing and able to engage socially, creating a positive feedback loop consistent with the “symptom-relationship vicious cycle” framework outlined earlier. Future research employing longitudinal mediation designs with repeated measures of potential mediators (e.g., self-efficacy, social skills) would be valuable in formally testing these hypothesized mechanisms.

However, this study has several limitations. First, single-center recruitment and a relatively small sample size may limit the generalizability of the findings. During the recruitment phase, 21 patients who met the inclusion criteria declined to participate due to personal reasons, and we did not systematically collect their baseline characteristics. This may introduce a certain degree of selection bias and further restrict the generalizability of the study results to a broader population. Future research should comprehensively record and report the basic characteristics of non-participants to evaluate the representativeness of the sample. Second, the assessment of friendship quality relied entirely on self-report measures, which may be subject to self-presentation bias and common method variance. Future studies could incorporate multi-method assessments, such as peer nominations, behavioral observations, or teacher/parent reports. Third, we did not systematically record the specific anxiety disorder subtypes (e.g., social anxiety disorder, generalized anxiety disorder) of the participants using a standardized diagnostic format. Although all participants met the ICD-10 diagnostic criteria for an anxiety disorder as confirmed by two attending psychiatrists, the lack of detailed subtype information precludes subgroup analyses that could examine whether SFGT exerts differential effects on friendship quality across different anxiety subtypes. This is particularly relevant given that adolescents with social anxiety disorder may face the most pronounced difficulties in peer relationships and social skills. Future studies should employ structured diagnostic interviews and systematically document anxiety subtypes to enable such analyses. Fourth, the control condition in this study consisted of a “non-interactive gathering” activity. While this design can control for some of the non-specific placebo effects associated with the setting, it cannot fully rule out peer interaction as a potentially positive factor. Therefore, the current results may represent a conservative estimate of the true effect size of SFGT compared with an active control, or may overestimate its effect compared with a passive control. Future studies could employ an attention-placebo control group with structured activities to further verify the unique contribution of Solution-Focused Group Therapy. Fifth, although the evaluators received training on maintaining blinding, participants may have naturally made references to group activities during the interviews, thereby providing cues that could allow the evaluators to infer group assignment. This potential risk has not yet been systematically controlled for. Future studies could consider adopting an independent video-based assessment approach to thoroughly eliminate such potential contamination. Sixth, although the intervention was delivered in a group format, the present analyses were conducted at the individual level using repeated-measures ANOVA and did not employ multilevel modeling to account for potential within-group nesting effects. Given the relatively small number of groups, the current analytical approach was retained; however, future studies with a larger number of groups should adopt multilevel modeling to appropriately account for the hierarchical structure of the data. Seventh, this study included adolescents aged 12–18 years in the same treatment groups. This age range spans multiple distinct developmental stages within adolescence, during which significant differences exist in friendship experiences, social needs, and cognitive maturity. These differences may have influenced group process dynamics and the heterogeneity of treatment effects. Due to the limited sample size, we were unable to conduct age-stratified analyses. Future studies should consider adopting narrower age bands or, with larger samples, exploring age as a potential moderator of treatment outcomes. Despite these limitations, the study employed methodologically rigorous designs—including randomization, blinded assessment, and longitudinal follow-upd longitudinal followignsort to the core conclusions (Javid et al., 2019).

In summary, the findings of this study suggest that Solution-Focused Group Therapy (SFGT) may be a promising and feasible intervention for simultaneously improving emotional symptoms and peer friendship quality in adolescents with anxiety disorders. This offers a potential new direction for adolescent mental health interventions—namely, leveraging group dynamics and a resource-oriented therapeutic framework to support their adaptation and growth in real-world social environments. Future research with larger, multi-center samples is needed to further explore the impact of SFGT on adolescents’ long-term social functioning and academic performance, which would facilitate the systematic application of this model in clinical and educational settings.

## Conclusion

This study investigated the effect of Solution-Focused Group Therapy (SFGT) on improving peer friendship quality among adolescents with anxiety disorders. The results suggest that this intervention may simultaneously alleviate anxiety and depressive symptoms while enhancing friendship quality, offering a potential pathway for disrupting the vicious cycle of mutually worsening symptoms and relationships within this population. From both theoretical and clinical practice perspectives, the findings provide preliminary support for the value of systematically integrating the interpersonal dimension into anxiety intervention frameworks. The short-term structured group protocol examined in this study represents a promising option that warrants further investigation and refinement in adolescent mental health services.

## Data Availability

The datasets presented in this article are not readily available because The data supporting the findings of this study involve personal health information of adolescent patients and are subject to ethical approval and patient confidentiality agreements. Therefore, the raw data are not publicly available to protect participant privacy. However, de-identified data can be made available from the corresponding author upon reasonable request and under a formal data use agreement that complies with ethical standards. Requests to access the datasets should be directed to Lixia Zhang, 45810281@qq.com.
