# Supplementary figures and images for: The effects of solution-focused group therapy on peer friendship quality in adolescents with anxiety disorders
Source: Front Psychol. 2026 Jun 10;17:1839700. doi: 10.3389/fpsyg.2026.1839700 (PMC13290894; doi:10.3389/fpsyg.2026.1839700)

## CONSORT 2010 flow diagram: Effects of SFGT on Adolescent Friendships Quality

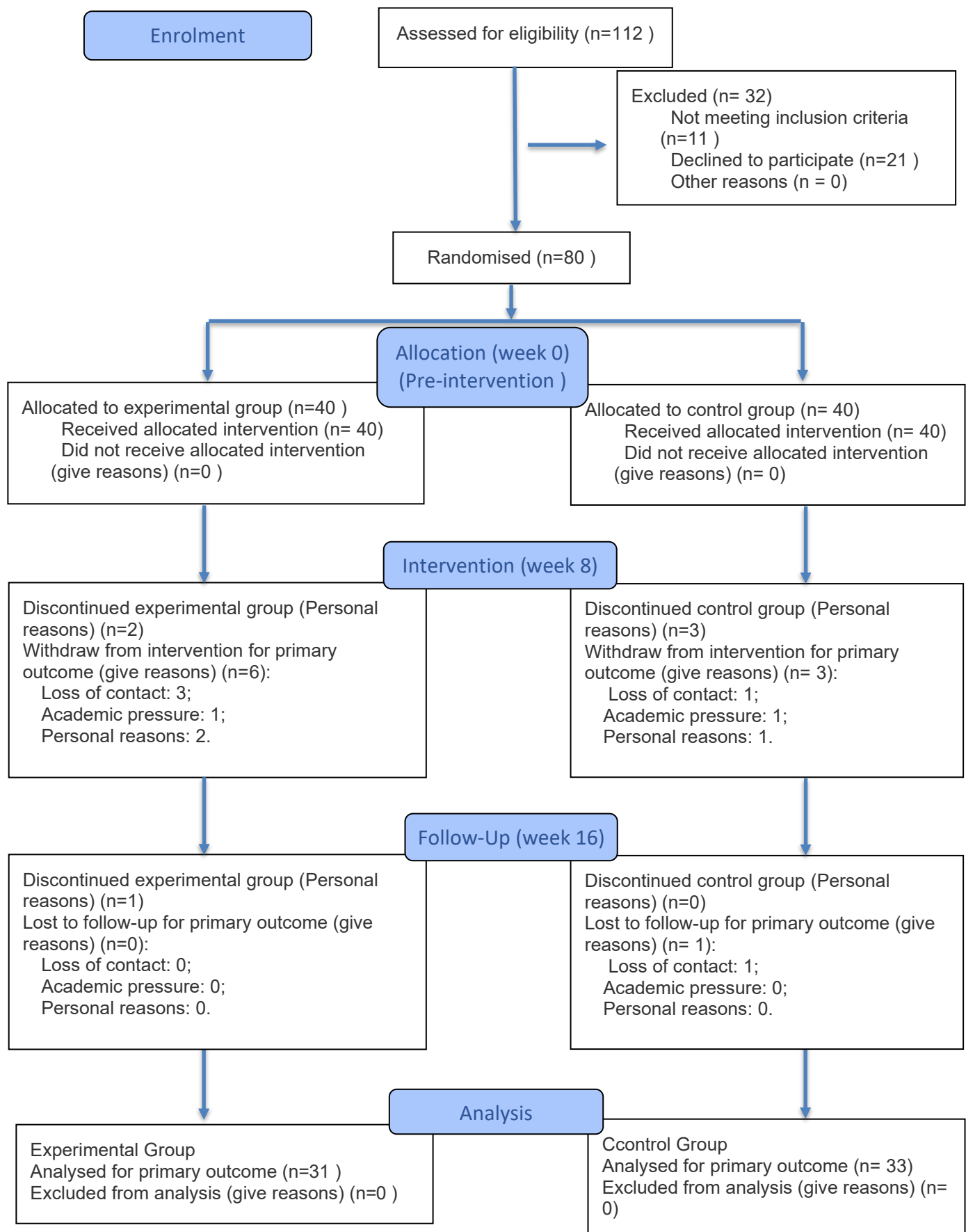

Supplement: Supplementary file 1 [file Data_Sheet_1.pdf]
