# Supplementary material for: The effects of solution-focused group therapy on peer friendship quality in adolescents with anxiety disorders
Source: Front Psychol. 2026 Jun 10;17:1839700. doi: 10.3389/fpsyg.2026.1839700 (PMC13290894; doi:10.3389/fpsyg.2026.1839700)
Supplement: Supplementary file 2 [file Table_1.pdf]

SFGT Session Overview

Unit No.	Session Theme	Main Activities	Solution-Focused Techniques
1	Nice to Meet You	1. Warm-up: Big Wind Blows — Members interact in a circle to ease strangeness.<br>2. Main activities: Name tag making (self-designed nickname badge and self-introduction); Snowballing Game — Retelling personal information of other members in groups.<br>3. Closing: Signing the group contract & sharing personal participation goals	Goal setting, Normalization
2	Cooperation and Friendship Growth	1. Warm-up: Squirrel and Tree; Whack-a-Mole — Team games to enhance cooperation and memorization among members.<br>2. Main activities: Homework review — Sharing interpersonal distress scenarios; Bag of Tricks — Discussing and proposing solutions in groups.<br>3. Closing: Intricate Knot Game — Holding hands to untangle the circle and strengthen mutual trust	Scaling questions, Exception-seeking questions, Compliments
3	Discovering Your Inner Strength	1. Warm-up: Guess Who He Is — Wearing name cards and guessing identity through	Concretization, Tracking questions,

Unit No.	Session Theme	Main Activities	Solution-Focused Techniques
		questioning.<br>2. Main activities: The Unique Orange — Observing individual features to analogize self-acceptance; Talent Showcase — Filling in strength inventory and sharing in groups.<br>3. Closing: Giving Compliments — Mutual praise to enhance self-confidence	Compliments
4	Communication With Each Other	1. Warm-up: Flower and Leaf grouping — Interactive exercise to improve listening ability.<br>2. Main activities: Dictation Drawing — Comparing one-way and two-way communication; Navigating the Minefield — Blindfolded guidance to build trust.<br>3. Closing: Mirroring Imitation — Action imitation to promote empathy	Snowballing, Scaling questions, First Sign
5	Conflict and Cooperation	1. Warm-up: Gentle Balloon Release — Activating group atmosphere.<br>2. Main activities: Boundary Setting — Learning to express personal needs through space defense; Rapid 60-second Card	Compliments, Scaling questions

Unit No.	Session Theme	Main Activities	Solution-Focused Techniques
		Sorting — Cultivating teamwork; Spaghetti Tower — Team building to improve creativity and collaboration.<br><br>3. Closing: Review and reflection on interpersonal conflict cases	
6	Dancing with Emotions	1. Warm-up: Name & Number Calling — Energetic interaction to introduce the theme of emotion.<br><br>2. Main activities: Inside Out video watching — Understanding underlying needs of emotions; Tree in the Rain painting — Exploring individual stress through artistic creation.<br><br>3. Closing: Sharing emotion regulation strategies	Exception-seeking questions, Hypothetical questions, Normalization
7	My Social World	1. Warm-up: Floating Balloons — Team interaction to enhance emotional bonding.<br><br>2. Main activities: Homework review — Sharing interpersonal changes; Social network sorting; OH Cards combined with WOOP strategy for goal visualization.<br><br>3. Closing: Sharing expectations	Compliments, Hypothetical questions

Unit No.	Session Theme	Main Activities	Solution-Focused Techniques
		for future growth	
8	Managing Separation & Growth	1. Warm-up: Rhythmic Clapping & Massage Exercise — Interactive relaxation to consolidate emotional connection.<br><br>2. Main activities: Homework review — Sharing gratitude expression; Sincere 100-second Eye Contact; Red String Web — Strengthening group collaboration.<br><br>3. Closing: Exchanging blessing cards to facilitate therapeutic separation and emotional closure	Normalization, EARS technique
